# Effects of salinity on upstream-migrating, spawning sea lamprey, *Petromyzon marinus*

**DOI:** 10.1093/conphys/cov064

**Published:** 2016-02-06

**Authors:** D. Ferreira-Martins, J. Coimbra, C. Antunes, J. M. Wilson

**Affiliations:** 1 Interdisciplinary Centre of Marine and Environmental Research (CIIMAR/CIMAR), University of Porto, Porto, Portugal; 2 Instituto de Ciências Biomédicas de Abel Salazar, ICBAS, Universidade do Porto, Porto, Portugal; 3 Aquamuseu do Rio Minho, Vila Nova de Cerveira, Portugal; 4 Department of Biology, Wilfrid Laurier University, Waterloo, Ontario, Canada

**Keywords:** Lamprey, osmoregulation, spawning migration

## Abstract

The sea lamprey, *Petromyzon marinus*, is an anadromous, semelparous species that is vulnerable to endangered in parts of its native range due in part to loss of spawning habitat because of man-made barriers. The ability of lampreys to return to the ocean or estuary and search out alternative spawning river systems would be limited by their osmoregulatory ability in seawater. A reduction in tolerance to salinity has been documented in migrants, although the underlying mechanisms have not been characterized. We examined the capacity for marine osmoregulation in upstream spawning migrants by characterizing the physiological effects of salinity challenge from a molecular perspective. Estuarine-captured migrants held in freshwater (FW) for ∼1 week (short-term acclimation) or 2 months (long-term acclimation) underwent an incremental salinity challenge until loss of equilibrium occurred and upper thresholds of 25 and 17.5, respectively, occurred. Regardless of salinity tolerance, all lamprey downregulated FW ion-uptake mechanisms [gill transcripts of Na^+^:Cl^−^ cotransporter (NCC/*slc12a3*) and epithelial Na^+^ channel (ENaC/*scnn1*) and kidney Na^+^/K^+^-ATPase (NKA) protein and activity but not transcript]. At their respective salinity limits, lamprey displayed a clear osmoregulatory failure and were unable to regulate [Na^+^] and [Cl^−^] in plasma and intestinal fluid within physiological limits, becoming osmocompromised. A >90% drop in haematocrit indicated haemolysis, and higher plasma concentrations of the cytosolic enzymes alanine aminotransferase, aspartate aminotransferase and lactate dehydrogenase indicated damage to other tissues, including liver. However, >80% of short-term FW-acclimated fish were able to osmoregulate efficiently, with less haemolysis and tissue damage. This osmoregulatory ability was correlated with significant upregulation of the secretory form of Na^+^:K^+^:2Cl^−^ cotransporter (NKCC1/*slc12a2*) transcript levels and the re-emergence of seawater-type ionocytes detected through immunohistochemical NKA immunoreactivity in the gill, the central ionoregulatory organ. This work sheds light on the molecular and physiological limits to the potential return to seawater for lampreys searching for alternative FW systems in which to spawn.

## Abstract (precis)

The molecular osmoregulatory abilities of the sea lamprey (anadromous, semelparous species), re-acclimated to saline conditions during the freshwater phase of its spawning migration have been studied. Short term freshwater acclimated lampreys can attain significant salinity tolerance whereas long term migrants cannot. The sea lamprey is a vulnerable species.

## Introduction

The sea lamprey *Petromyzon marinus* (Linnaeus 1758) has an anadromous life history that is characterized by three distinctive stages (Hardisty and Potter, 1971a,b; Beamish, 1980a). During the ammocoete larval stage, sea lampreys are freshwater (FW) benthic stream filter feeders, after which they undergo a dramatic morphological and physiological transformation into parasitically feeding juveniles that migrate to the ocean, where they become parasitic feeders. The adults re-enter freshwater, migrating upstream until they find a suitable place for terminal spawning. In Europe, sea lamprey populations are declining and facing the threat of extinction as a result of overharvesting of adults and physical loss of spawning and nursery grounds because of construction of man-made barriers (dams and weirs) blocking access to suitable upstream spawning grounds as well as habitat destruction (Renaud, 1997; Almeida *et al.*, 2002b; Close *et al.*, 2002). An understanding of the physiological limitations to cope with these challenges is vital to the management of these threatened or endangered lampreys.

Lampreys are osmoregulators, and their spawning migration requires a switch from marine hyposmoregulation to FW hyperosmoregulation (Beamish, 1980b). The gill, the kidney and the intestine are the primary organs involved in the active regulation of internal levels of ions using mechanisms proposed to be similar to those of teleost fishes (Morris, 1972; Beamish, 1980b; Hardisty *et al.*, 1989; Bartels and Potter, 2004). In marine environments, fishes drink seawater and excrete excess ions across their gills using seawater-type ionocytes to compensate for osmotic water losses and passive ion gains, respectively (Evans *et al.*, 2005; Marshall and Grosell, 2006). In contrast, in FW fishes actively take up ions using freshwater-type ionocytes to compensate for passive ion losses and produce copious amounts of dilute urine to get rid of osmotically gained water (Evans *et al.*, 2005; Marshall and Grosell, 2006).

The branchial mechanism of NaCl secretion by seawater ionocytes in lampreys is likely to be by the well-characterized secondary active Cl^−^ secretion mechanism (Bartels and Potter, 2004; Evans *et al.*, 2005). Basolateral Na^+^/K^+^-ATPase creates a favourable electrochemical gradient for Cl^−^ to enter the cell via Na^+^:K^+^:2Cl^−^ cotransporter 1 (NKCC1/*slc12a2*) and out apically though a channel homologous to the cystic fibrosis transmembrane conductance regulator (CFTR/*abcc7*; Singer *et al.*, 1998; Wilson *et al.*, 2000; Marshall *et al.*, 2002b). Sodium ions accumulate extracellularly and leak out paracellularly through leaky tight junctions associated with these ionocytes (Smith, 1930; Karnaky, 1980, 1986; Loretz, 1995).

In the FW lamprey gill, Na^+^ uptake is predicted to be mediated by the epithelial Na^+^ channel (ENaC/*scnn1*) through indirect coupling with the electrogenic vacuolar H^+^-ATPase pump (V-ATPase; Bartels and Potter, 2004). Teleost fishes lack the ENaC and instead Na^+^ uptake is via an acid sensing ion channel Na^+^ channel (ASIC) (Dymowska *et al.*, 2014). A pool of intracellular H^+^ ions for the V-ATPase is produced by CO_2_ hydration catalysed by carbonic anhydrase (CA), and low intracellular Na^+^ levels are maintained by basolateral Na^+^/K^+^-ATPase. Chloride uptake is likely to be performed by epithelial cells with a Cl^−^ channel in the basolateral membrane, cytosolic CA (Larsen, 1991) and an apical Cl^−^/HCO_3_ antiport system (Garcia-Romeu and Ehrenfeld, 1975; Larsen, 1991; Marshall *et al.*, 1997). Immunohistochemistry of V-ATPase and CA support branchial mitochondrion-rich intercalated cells as likely ionocytes (Choe *et al.*, 2004; Reis-Santos *et al.*, 2008).

Drinking rates have been measured in lampreys, and when in seawater lampreys swallow from 5 to 99 ml kg^−1^ day^−1^, which is desalinized and absorbed by the gut (Pickering and Morris, 1970). During acclimation to freshwater, drinking decreases to very low levels (Pickering and Morris, 1970; Rankin, 2002). The mechanism of absorption of water across the intestine is likely to involve an apical Na^+^:K^+^:2Cl^−^ cotransporter (NKCC2/*slc12a1*) and Na^+^:Cl^−^ cotransporter (NCC/*slc12a3*) and to be driven by basolateral Na^+^/K^+^-ATPase (Cutler *et al.*, 1996; Cutler and Cramb, 2001; Marshall *et al.*, 2002a).

The anadromous sea lamprey’s upstream spawning migration can be divided into the following three distinct stages: (i) migration from the ocean to the estuary; (ii) pre-spawning holding in the estuary; and (iii) upstream movement within rivers and streams to spawning sites (Applegate, 1950; Clemens *et al.*, 2010). Given that barriers to progress on the spawning migration of lampreys present a potential conservation issue (Renaud, 1997; Close *et al.*, 2002; Lucas *et al.*, 2009), the ability of migrants, which have no fidelity to natal streams (Moser *et al.*, 2015), to search out alternative rivers for spawning may be significant (Kelso and Gardner, 2000; Noyes *et al.*, 2013; Holbrook, 2015). However, in a number of studies it has been found that approximately half-strength seawater is lethal to upstream-migrating anadromous lampreys (Galloway, 1933; Morris, 1956; Beamish *et al.*, 1978). Specifically, Beamish *et al*. (1978) determined the upstream migrant sea lamprey 48 h LC_50_ (lethal concentration to kill 50%) salinity to be ∼15.2.

To date, our understanding of physiological constraints in lampreys is poorly studied and understood, especially at the molecular level (Moser *et al.*, 2015). Thus, the aim of the present study was to determine the physiological effects of salinity challenge in estuarine migrating adult sea lamprey after short- (∼1 week) and long-term (∼2 months) acclimation to FW. The study was focused on the molecular and physiological changes in the gill, kidney and intestine, in addition to a number of osmoregulatory end points in plasma and muscle. In short-term FW-acclimated salinity-challenged fish, osmoregulators and osmocompromised groups were identified by changes in plasma Na^+^ and Cl^−^ ions and haematocrit and subsequently analysed as separate groups.

## Materials and methods

### Animals

Freshwater migrating adult sea lampreys were collected by artisanal fishermen at the mouth of the River Minho estuary (41.874546 N, −8.849831 W; Araújo *et al*., 2013) using drift trammel nets (Quintella, 2006) during the spring of 2012 and 2013 for the short- and long-term FW-acclimation studies, respectively. The River Minho has a salt wedge type estuary (presence of a vertical halocline) with a salinity ranging from 7 to 33 and temperature ∼14°C. The animals were held in the Aquamuseu in Vila Nova Cerveira in flow-through FW tanks and then transported to the Interdisciplinary Centre of Marine and Environmental Research (CIIMAR) and maintained in 1000 l tanks with recirculated (mechanical and biological filtration) dechlorinated Oporto city tap water at 16°C. Animals were not fed because they do not feed during this stage of their life cycle (Larsen, 1980). To minimize the effects of handling stress during experimentation, animals were acclimated to these tank conditions for at least 1 week before experimentation. Salinity, pH, dissolved oxygen and temperature were monitored daily using a multiparameter analyser (HQ40d; Hach Lange, Loveland, CO, USA). Total ammonia was monitored every third day using a commercial kit (04910-NH4/NH3-Test; Sera GmbH, Germany). Animals were treated in accordance with the Portuguese Animal Welfare Law (Decreto-Lei no. 197/96) and animal protocols approved by CIIMAR/Universidade do Porto and Direção-Geral de Alimentação e Veterinária (Ministry of Agriculture).

### Experimental procedure for long-term freshwater acclimation salinity challenge

For the first experiment, conducted from May to June 2012, a total of eight sea lampreys (76.4 ± 1.4 cm total length and 840.7 ± 46.3 g wet mass) were acclimated to the 1000 l water system with dechlorinated tap water at 16°C. Two groups of four lamprey were then transferred to two 80 l tanks with a renewal water flow rate of 6 l min^−1^. In one of the tanks, salinity was increased by 5 every 2 days using natural, filtered seawater at 35 until a brackish water (BW) salinity of 15 was reached. From this point on, salinity was increased by 2.5 every following day to a final salinity of 17.5 (total of 8 days). Preliminary trials indicated this to be a salinity limit. The second tank was set for the control animals held in FW that were also sampled at the end of the experiment. Water parameters and mortalities were monitored daily.

### Experimental procedure for short-term freshwater acclimation salinity challenge

For the second experiment, conducted from March to April 2013, a total of 30 sea lampreys (78.1 ± 0.8 cm total length and 855.3 ± 20.3 g wet mass) were divided into five group tanks with six lampreys in each. The same experimental procedure as in the first experiment was carried out in four of the tanks until a final salinity of 25 was reached (total of 11 days). Preliminary trials established this as the salinity limit. Six animals were randomly sampled at a salinity of 17.5 [determined upper salinity limit for the first experiment and similar to the limit reported by Beamish *et al*. (1978), Galloway (1933) and Morris (1956)] and the remainder at 25. The fifth tank was set as the control group and held in FW, and fish were sampled at the beginning and end of the experimental period (*n* = 3 + 3). No differences were detected between the beginning and the end, so the control group data were merged.

### Sampling

Animals were anaesthetized with 2-phenoxyethanol (1:2000) and killed by cervical transection. Total fish length (in millimetres) and mass (±0.01 g) were measured, gender was determined (presence of testes or ovaries) and Fulton’s condition factor was calculated (Fulton, 1902; Ricker, 1975). Blood samples were collected from the caudal vessel using a sterile syringe coated with lithium heparin (Sigma-Aldrich, St. Louis, MO) and centrifuged at 13 000***g*** for 3 min at room temperature (Pico 17; Heraeus, Harau, Germany). Haematocrit was measured in duplicate to the nearest millimetre and converted to percentage of total blood volume. Plasma and red blood cells were separated, snap frozen in liquid nitrogen and stored at −80°C. The peritoneal cavity was opened ventrally and the gut ligated at anterior and posterior extremities and removed. Gut fluid was collected by draining gut content into Falcon tubes, centrifuged at 13 000***g*** for 3 min at room temperature, and the supernatant was used to quantify ion concentrations. Gill, kidney, anterior, middle and posterior intestine samples were also collected, and all tissue samples were snap frozen in liquid nitrogen and stored at −80°C for further use. One gram of epaxial muscle tissue was also collected to determine water content and Na^+^ and K^+^ concentrations. A gill pouch was fixed using 10% neutral buffered formalin at 4°C for 24 h and stored in 70% ethanol at 4°C for paraffin embedding. For measurement of Na^+^/K^+^-ATPase activity, gill samples were also collected as described by McCormick (1993).

### Ion quantification

Muscle samples were dried to constant mass at 60°C for determination of water content. Dried muscle was then digested in five volumes of 65% nitric acid for 3 days. The Na^+^ and K^+^ concentrations were quantified using a flame photometer (model PFP7; Jenway, Felsted, UK) as performed by Wilson *et al*. (2007b). Plasma and gut fluid samples were also analysed. Chloride concentration was measured in gut fluid and plasma samples by titration (Chloride Analyzer 925; Corning, Halstead, UK).

### Isolation and quantification of RNA and synthesis of complementary DNA

Total RNA was extracted using Aurum™ Total RNA Mini Kits according to the manufacturer’s recommendations (Bio-Rad, Hercules, CA, USA). Homogenization was done in a bead mill (Precellys 24; Bertin Technologies, Montigny-le-Bretoneux, France) at 6400 rpm for two cycles of 15 s with 5 s interval. Homogenates were centrifuged for 2 min at 14 000 *g* at room temperature (Eppendorf MiniSpin Plus, Hamburg, Germany). On-column DNaseI treatment was performed. Total RNA concentration and purity were assessed using a Nanodrop spectrophotometer (Thermo Scientific, Wilmington, DE, USA) and integrity was determined by agarose gel electrophoresis (Bio-Rad) in 1.2% formaldehyde agarose gels stained with GelRed (Biotium, Hayward, CA, USA). Total RNA samples were stored at −80°C. The cDNA was synthesized from 1 μg of total RNA with iScript cDNA Synthesis Kit in a 20 µl reaction volume (Bio-Rad). Reactions were carried out in a Doppio thermocycler (VWR International Ltd, Lisbon, Portugal) at 25°C for 5 min; 42°C for 30 min; and 85°C for 5 min. Samples were stored at −20°C.

### RT-PCR and RT real-time PCR

The PCRs were performed using 0.4 µl sample cDNA, 2 mM MgCl_2_, 0.2 mM dNTPs, 0.5 µM of each primer and 0.025 U GoTaq^®^ DNA polymerase (Promega, Madison, WI, USA) and 4 µl of 5× Green GoTaq^®^ reaction buffer, respectively, in 20 µl reaction volumes. Primers were designed using Primer3 (Rozen and Skaletsky, 2000) and were initially tested for specificity by RT-PCR. Reactions consisted of an initial denaturation at 94°C for 30 s followed by 35 cycles of: 94°C for 30 s; annealing at 58 or 60°C for 30 s; extension at 72°C for 30 s; and ending with a final extension for 2 min at 72°C.

The PCR products were separated on 2% agarose TBE (Tris-borate-EDTA) gels at 80 V to confirm the size of amplicons. All gels were stained with GelRed and images acquired with a Fujifilm LAS-4000 Mini luminescent image analyzer (Fujifilm, Tokyo, Japan).

Relative levels of mRNAs for epithelial sodium channel (*scnn1*/ENaC), sodium/potassium ATPase α1-subunit (*atp1a1*/NKA-a), vacuolar-type H^+^-ATPase (*atp6v1*E/V-ATPase E), sodium:potassium:chloride cotransporter 1 (*slc12a2*/NKCC1), sodium:chloride cotransporter (*slc12a3*/NCC) and corticosteroid receptor (*cr*) genes were quantified by real-time RT-PCR analysis using SYBR green with an iQ5 Multicolor Real-Time PCR Detection System (Bio-Rad). Each cDNA sample was diluted 50 times and then 5 µl added to a reaction mix containing 10 µl of 2× iQ SYBR Green Supermix (Bio-Rad) and 250 nM of each primer in a total volume of 20 µl. The cycle profile was as follows for the given primers pairs: 95°C for 3 min, followed by 40 cycles of 95°C for 10 s, 58 or 60°C (see supplemental table 1) for 30 s and 72°C for 30 s. A melt curve was generated for every PCR product to confirm the specificity of the assays and a dilution series was prepared to check the efficiency of the reactions. The *gapdh* was used as the housekeeping gene. The comparative CT method (2^−ΔΔCT^ method) based on cycle threshold (CT) values was used to analyse the expression levels of the genes of interest. Random resulting amplicons were run on 2% agarose TBE gel to confirm the presence of a single amplified product of the expected size.

### Immunofluorescence microscopy

Immunofluorescence localization of NKA α-subunit and V-ATPase B subunit were performed according to Wilson *et al*. (2007a) using a double labelling protocol (Wilson *et al.*, 2007a; Reis-Santos *et al.*, 2008). The antibodies against NKA and V-ATPase were the α5 mouse monoclonal and rabbit BvA1 polyclonal antibodies (Takeyasu *et al.*, 1988; Wilson *et al.*, 2007b).

### Plasma lactate, lactate dehydrogenase, alanine aminotransferase and aspartate aminotransferase

Determinations of plasma lactate, lactate dehydrogenase (LDH), alanine aminotransferase (ALT) and aspartate aminotransferase (AST) were performed using commercial kits according to manufacturer’s instructions (ref nos 1001330, 41220, 1001170 and 1001160, respectively; Spinreact, Sant Esteve d’en Bas, Spain).

### Measurement of gill Na^+^/K^+^-ATPase activity

The Na^+^/K^+^-ATPase activity was measured via a kinetic microassay at 25°C (McCormick, 1993; Reis-Santos *et al.*, 2008) using a BioTek Synergy 2 microplate reader (BioTek Instruments; Winooski, VT, USA) and Gen5™ reader control and data analysis software (Gen5; BioTek Instruments). Samples stored in 300 µl SEI buffer were thawed on ice, sodium deoxycholate was added to a final concentration of 0.1%, and samples were homogenized using a Precellys 24 bead mill. Homogenates were centrifuged at 14 000***g*** for 5 min at 4°C and the supernatants decanted and used for the ATPase assay and immunoblotting experiments. Samples of 10 µl were run in two duplicate sets. In one set, ouabain (1.0 mmol l^–1^) was added to the assay mixture specifically to inhibit Na^+^/K^+^-ATPase activity. Total protein was measured by Bradford’s method (Bradford, 1976) using bovine serum albumin as a standard.

### Immunoblotting

The unused supernatant from Na^+^/K^+^-ATPase activity assay was mixed with an equal volume of 2× Laemmli’s buffer (Laemmli, 1970), heated for 10 min at 70°C and then stored at 4°C. Protein concentrations were adjusted to 1 µg µl^−1^ using 1× Laemmli’s buffer. Immunoblotting was performed as described by Reis-Santos *et al*. (2008). Blots were probed with mouse anti-β-actin monoclonal (1:500; Sigma-Aldrich) and αRbNKA (1:1000; α-subunit of the Na^+^/K^+^-ATPase) antibodies and signal was obtained by enhanced chemiluminescence (ECL) with Millipore Immobilon Western chemiluminescent HRP substrate (Millipore Corporation, Billerica, MA, USA). Images were acquired using a luminescent image analyser (Fujifilm LAS-4000 mini) and image reader software (LAS-4000 version 2.0). The intensity of the band signal was quantified using an image analysis software program (Multi Gauge v3.1; Fujifilm).

### Statistical analysis

Statistical differences between groups were determined using one-way ANOVA followed by the *post hoc* Student–Newman–Keuls test. Comparisons between the effect of salinity on different tissues were performed using a two-way ANOVA followed by a Holm–Sidak method pairwise multiple comparison (SigmaPlot 11.0; Systat Software, Inc.). Data are shown as means ± SEM. The fiducial limit was set at 0.05.

## Results

### Haematocrit and leukocrit

In long-term FW-acclimated lampreys, haematocrit values decreased by 98% with salinity (17.5) challenge, whereas in the short-term FW-acclimated lampreys at the same salinity levels the haematocrits decreased by only 23% (Table 1). Short-term FW-acclimated lampreys challenged with BW-25 which were osmoregulating had a similar 21% decrease, whereas in osmocompromised fish the haematocrit decreased by 93%. In response to salinity, leukocrit levels increased in long-term FW-acclimated lampreys challenged to BW-17.5. In contrast, in short-term FW-acclimated lampreys the leukocrit decreased in BW-25 osmoregulators (Table 1).

**Table 1:** Gender (male:female), total length (in centimetres), mass (in grams), Fulton’s condition (K) factor, haematocrit (as a percentage) and leukocrit (as a percentage) in long-term freshwater (FW)-acclimated sea lampreys challenged in FW (*n* = 4) and 17.5 brackish water (BW-17.5; *n* = 4) and short-term FW-acclimated sea lampreys challenged in FW (*n* = 6), BW-17.5 (*n* = 6), and 25 brackish water (BW-25) osmoregulators (*n* = 14) and BW-25 osmocompromised animals (*n* = 4)

FW acclimation	Group	Gender (male:female)	Length (cm)	Mass (g)	Fulton’s K factor	Haematocrit (%)	Leukocrit (%)
Long term	FW	2:2	74.6 ± 4.2	758.1 ± 69.3	1.94 ± 0.29	36.7 ± 1.5^a^	2.1 ± 0.1^a^
	BW-17.5	2:2	78.1 ± 3.5	923.3 ± 130.8	1.83 ± 0.18	0.6 ± 0.33^b^	3.8 ± 0.7^b^
Short term	FW	3:3	78.6 ± 0.5	892.2 ± 18.6	1.84 ± 0.05	45.2 ± 1.2^a^	2.4 ± 0.3^a^
	BW-17.5	2:4	78.5 ± 1.5	873.0 ± 44.7	1.81 ± 0.06	34.8 ± 0.5^b^	1.6 ± 0.3^ab^
	BW-25 osmoregulating	6:8	78.5 ± 0.9	859.5 ± 25.7	1.77 ± 0.37	35.7 ± 0.9^b^	1.5 ± 0.1^b^
	BW-25 osmocompromised	3:1	78.4 ± 2.4	845.4 ± 14.7	1.78 ± 0.13	3.1 ± 1.1^c^	1.8 ± 0.2^ab^

Values are shown as means ± SEM. Within each experiment, groups that do not share letters are significantly different (*P* < 0.05).

### Ion concentrations in plasma, muscle and intestinal fluid

Plasma Na^+^ and Cl^−^ concentrations increased significantly in all salinity-challenged animals in both experiments. Nonetheless, only long-term FW-acclimated sea lampreys challenged to BW-17.5 and osmocompromised short-term FW-acclimated lampreys challenged to BW-25 showed increases in these ions that approached those levels found in the environment. Plasma Ca^2+^ concentrations increased 3-fold in long-term FW-acclimated salinity-challenged migrants and 10-fold in short-term FW-acclimated salinity-challenged osmocompromised animals at BW-25. Plasma K^+^ levels remained unaltered in long-term FW-acclimated salinity-challenged animals, and a decrease was observed in short-term FW-acclimated salinity-challenged BW-25 osmocompromised animals (Table 2).

**Table 2:** Plasma Na^+^, K^+^, Ca^2+^ and Cl^−^ concentrations in long-term freshwater (FW)-acclimated sea lampreys challenged in FW (*n*= 4), 17.5 brackish water (BW-17.5; *n* = 4) and short-term FW-acclimated sea lampreys challenged in FW (*n* = 6), BW-17.5 (*n* = 6), and 25 brackish water ’(BW-25) osmoregulators (*n* = 14) and BW-25 osmocompromised animals (*n* = 4)

FW acclimation	Group	[Na^+^]	[K^+^]	[Ca^2+^]	[Cl^−^]
Long term	FW	137.7 ± 1.1^a^	3.2 ± 0.3	1.8 ± 0.5^a^	96.5 ± 0.8^a^
	BW-17.5	203.3 ± 1.3^b^	3.0 ± 0.4	5.8 ± 0.5^b^	201.3 ± 6.6^b^
Short term	FW	151.3 ± 3.8^a^	5.5 ± 0.5^a^	2.8 ± 0.3^a^	108.1 ± 1.4^a^
	BW-17.5	189.2 ± 3.9^b^	4.0 ± 0.2^ab^	4.8 ± 0.1^ab^	141.0 ± 5.6^b^
	BW-25 osmoregulating	195.0 ± 8.6^b^	4.6 ± 0.1^ab^	4.9 ± 0.3^ab^	148.6 ± 6.7^b^
	BW-25 osmocompromised	288.7 ± 3.7^b^	3.3 ± 0.1^b^	25.9 ± 2.8^b^	275.5 ± 15.7^c^
FW		0.5	0.1	0.6	0.1
BW-17.5		237.2	5.4	4.9	286.1
BW-25		350.8	7.3	8.1	421.0

Values are shown as means ± SEM. Within each experiment, groups that do not share letters are significantly different (*P* < 0.05). Corresponding water ion concentrations are also listed.

Muscle Na^+^ levels were 37-fold higher in long-term FW-acclimated salinity-challenged migrants. For the short-term FW-acclimated salinity-challenged upstream-migrating groups, no differences from the FW control group were observed, although in the BW-25-acclimated animals there was a significant difference between osmoregulators and osmocompromised fish (Table 3). Potassium levels were higher in BW-17.5 sea lampreys from the long-term FW-acclimated salinity-challenge experiment, whereas animals at the same salinity in the short-term FW-acclimated salinity-challenge experiment registered a decrease. The Na^+^:K^+^ ratio was only increased in BW-17.5 animals from the long-term FW-acclimated salinity-challenge experiment. Muscle water content in short-term FW-acclimated lampreys decreased in BW-17.5 animals, and levels decreased further in both BW-25 groups. Intestinal fluid [Cl^−^] increased in long-term FW-acclimated salinity-challenged lampreys, approaching environmental levels. The same effect was observed in the BW-25 osmocompromised sea lampreys from the short-term FW-acclimation experiment.

**Table 3:** Muscle concentrations of Na^+^ and K^+^ (in millimoles per gram wet mass), percentage water and Na^+^:K^+^ ratio and the intestinal fluid Cl^−^ concentration (millimolar) in long-term freshwater (FW)-acclimated sea lampreys challenged in FW (*n* = 4), 17.5 brackish water (BW-17.5; *n* = 4) and short-term FW-acclimated sea lampreys challenged in FW (*n* = 6), BW-17.5 brackish water (*n* = 6), and 25 brackish water (BW-25) osmoregulators and BW-25 osmocompromised animals (*n* = 4)

FW acclimation	Group	Muscle				Intestinal fluid
		[Na^+^]	[K^+^]	Water content	Na^+^:K^+^ ratio	[Cl^−^]
Long term	FW	23.8 ± 5.1^a^	85.2 ± 17.4^a^	77.1 ± 0.4	0.28 ± 0.01^a^	114.8 ± 0.1^a^
	BW-17.5	889.0 ± 13.4^b^	179.2 ± 30.2^b^	75.3 ± 0.9	0.51 ± 0.07^b^	243.5 ± 2.0^b^
Short term	FW	58.6 ± 8.7^ab^	93.0 ± 8.1^a^	74.4 ± 1.4^a^	0.63 ± 0.07	117.9 ± 28.7^a^
	BW-17.5	42.3 ± 4.8^ab^	56.9 ± 5.2^b^	68.9 ± 1.0^b^	0.74 ± 0.02	109.7 ± 9.5^a^
	BW-25 osmoregulating	36.4 ± 5.0^b^	73.2 ± 5.7^ab^	63.9 ± 0.6^c^	0.50 ± 0.06	121.4 ± 5.1^a^
	BW-25 osmocompromised	73.9 ± 5.4^ac^	93.8 ± 12.3^a^	62.1 ± 0.8^c^	0.84 ± 0.14	233.9 ± 26.9^b^

Values are shown as means ± SEM. Within each experiment, groups that do not share letters are significantly different (*P* < 0.05).

### Plasma lactate, lactate dehydrogenase, alanine aminotransferase and aspartate aminotransferase

Plasma lactate concentrations and activities of LDH, ALT and AST are presented in Table 4. In BW-17.5 long-term FW-acclimated salinity-challenged sea lampreys, plasma lactate decreased ∼3-fold. However, in short-term FW-acclimated sea lampreys, no differences in plasma lactate were observed except for a significant difference between BW-25 osmoregulators and osmocompromised fish. Plasma LDH activity was significantly higher (∼176-fold) only in animals from the short-term FW-acclimation experiment BW-25 osmocompromised group compared with the FW control. High variability was found in LDH activities in the BW-17.5 challenged animals in the long-term FW-acclimated lamprey challenge experiment, and thus, the difference was not significant. Plasma ALT activity was significantly elevated in long-term FW-acclimated BW-17.5 lampreys and short-term FW-acclimated BW-25 osmocompromised animals. Plasma AST activity did not show a change in long-term FW-acclimated sea lamprey groups, although high variation was observed in the BW-17.5 group. In short-term FW-acclimated BW-25 osmocompromised animals, AST activity was significantly elevated.

**Table 4:** Lactate, lactate dehydrogenase (LDH), alanine aminotransferase (ALT) and aspartate aminotransferase (AST) in long-term freshwater (FW)-acclimated sea lampreys challenged in FW (*n* = 4), 17.5 brackish water (BW-17.5; *n* = 4) and short-term FW-acclimated sea lampreys challenged in FW (*n* = 6), BW-17.5 (*n* = 6), and 25 brackish water (BW-25) osmoregulators (*n* = 14) and BW-25 osmocompromised animals (*n* = 4)

FW acclimation	Group	Lactate (mM)	LDH (U l^−1^)	ALT (U l^−1^)	AST (U l^−1^)
Long term	FW	8.12 ± 0.43^a^	172.6 ± 57.2	0 ± 2.0^a^	165.1 ± 10.5
	BW-17.5	2.73 ± 0.51^b^	2983.7 ± 1179.7	123.0 ± 57.4^b^	600.1 ± 246.7
Short term	FW	2.00 ± 0.45^ab^	8.5 ± 0.8^a^	1.9 ± 1.4^a^	61.2 ± 1.6^a^
	BW-17.5	1.84 ± 0.44^ab^	10.3 ± 2.2^ab^	4.5 ± 0.3^ab^	103.7 ± 20.5^ab^
	BW-25 osmoregulating	1.00 ± 0.14^a^	13.0 ± 1.5^ab^	3.4 ± 0.9^ab^	94.8 ± 12.9^ab^
	BW-25 osmocompromised	5.65 ± 3.48^b^	1493.9 ± 1334.1^b^	15.6 ± 2.6^b^	351.6 ± 125.8^b^

Values are shown as means ± SEM. Within each experiment, groups that do not share letters are significantly different (*P* < 0.05).

### Na^+^/K^+^-ATPase activity

**Figure 1:**
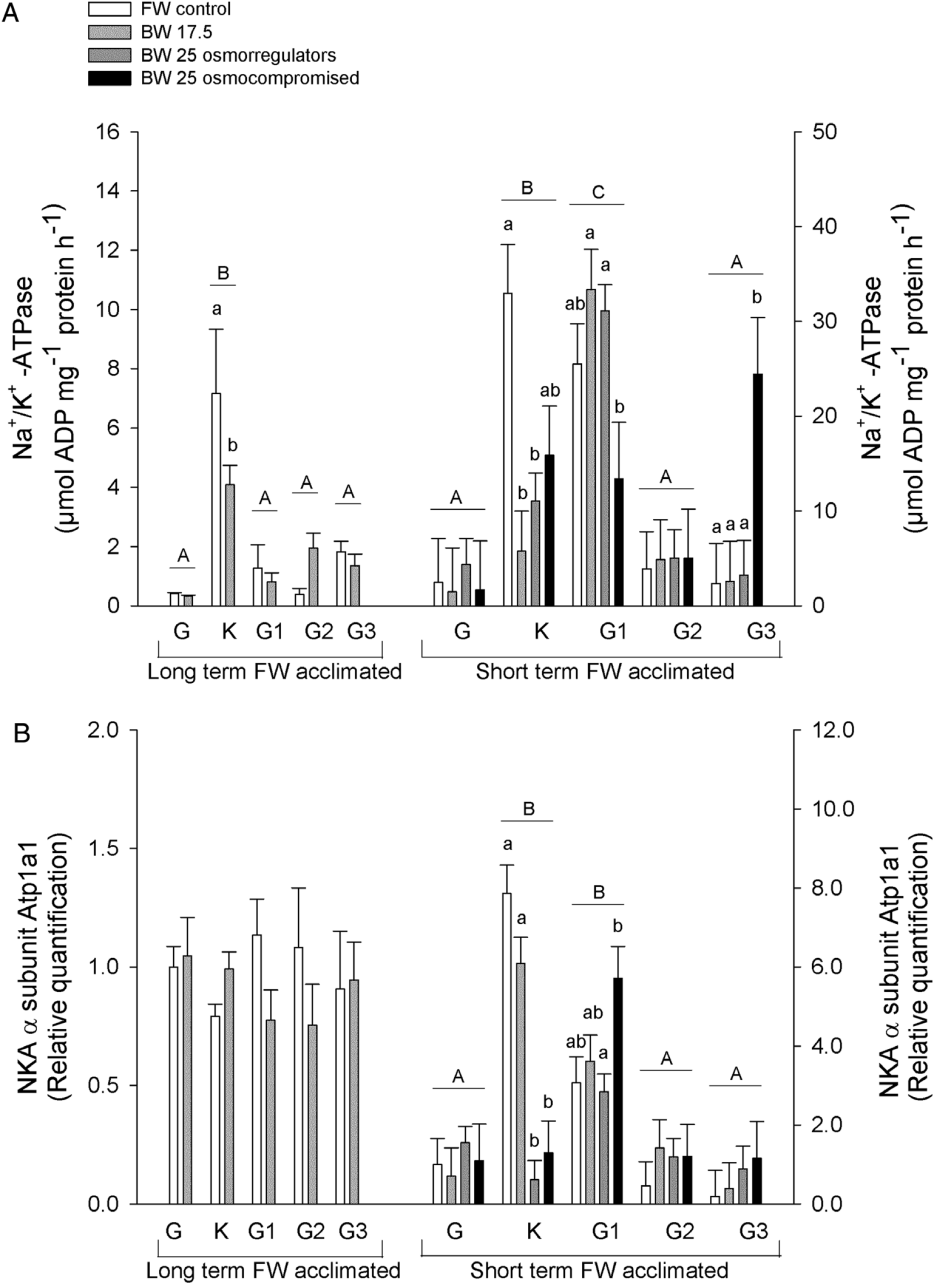
(**A**) Na^+^/K^+^-ATPase activity in the following tissues of long- and short-term freshwater (FW)-acclimated adult sea lampreys exposed to salinities (BW) of 17.5 and 25: G, gill; K, kidney; G1, anterior intestine; G2, middle intestine; and G3, posterior intestine. Short-term FW-acclimated sea lamprey freshwater control groups, FW control (*n* = 6, except gill and kidney *n* = 5 and middle intestine *n* = 8); brackish water 17.5 acclimated groups, BW-17.5 (*n* = 6, except anterior intestine *n* = 7); and brackish water 25, which are subdivided into osmoregulating and osmocompromised animals: BW-25 osmoregulating (*n* = 14, except kidney *n* = 13, anterior and posterior intestine *n* = 12 and *n* = 8, respectively); and BW-25 osmocompromised (*n* = 4, except posterior intestine *n* = 3). All long-term FW-acclimated sea lampreys tissue groups were *n* = 4. (**B**) Representative expression of NKA α1-subunit, Atp1a1 (1:1000). Values are relative to the respective freshwater control. Values are shown as means + SEM. Different upper case letters indicate significant differences between tissues irrespective of salinity. Different lower case letters denote significant differences with salinity within each tissue. Analysis was performed using a two-way ANOVA followed by a Holm–Sidak method test; *P* < 0.05.

Gill Na^+^/K^+^-ATPase activities are presented in Fig. 1A. In the long-term FW-acclimated sea lampreys, Na^+^/K^+^-ATPase activity was highest in the kidney, which decreased from approximately 7.2 to 4.1 µmol ADP (mg protein)^−1^ h^−1^ when animals were acclimated to BW-17.5. Short-term FW-acclimated sea lampreys challenged to BW-17.5 and the BW-25 osmoregulators also displayed a decrease in NKA activity in the kidney of approximately 7- and 3.6-fold, respectively. The BW-25 osmocompromised animals displayed a tendency to retain higher NKA activity levels, because levels were not different from the FW group.

In short-term FW-acclimated lampreys, NKA activity was highest in the anterior intestine. In BW-25 osmocompromised animals, NKA activity was significantly lower than in the other salinity-challenged groups but not in the FW control. In contrast, in the posterior intestine of short-term FW-acclimated lampreys, the BW-25 osmocompromised animals had significantly higher NKA activity than the other groups. No differences in NKA activities in the intestinal regions in long-term FW-acclimated lampreys were observed. Also, in neither experiment were gill NKA activities altered by salinity challenge.

### Immunoblotting

Immunoblotting analyses of NKA α-subunit show no changes of protein expression in the long-term FW-acclimated sea lampreys; however, in the short-term FW-acclimated lampreys, higher expression was found in the kidney and anterior intestine. In the kidney, expression was lower in both BW-25 groups. In contrast, in the anterior intestine, NKA protein levels did not change compared with FW control animals; however, BW-25 osmocompromised animals had significantly greater levels than osmoregulators (Fig. 1B).

### Real-time RT-PCR

**Figure 2:**
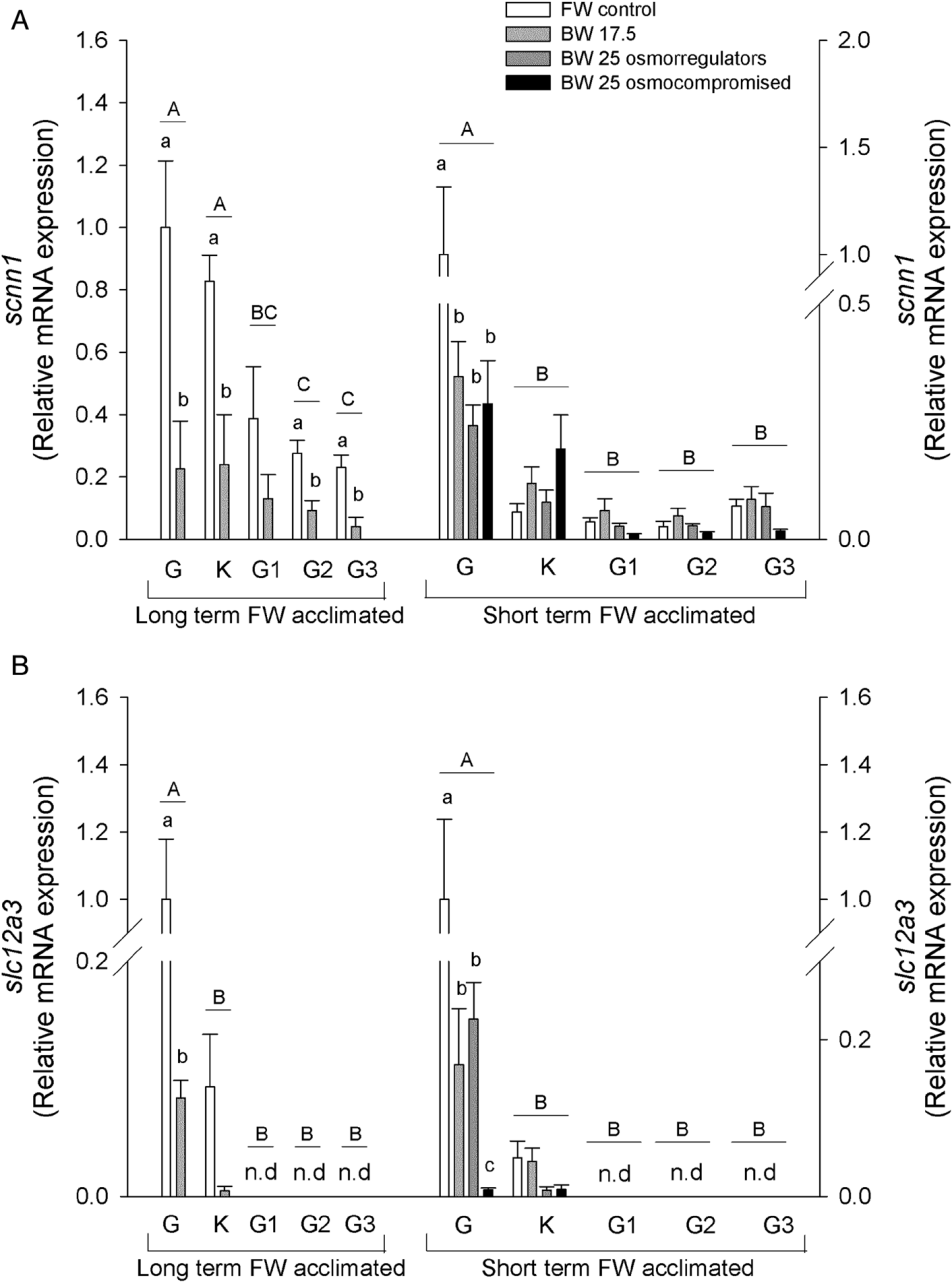

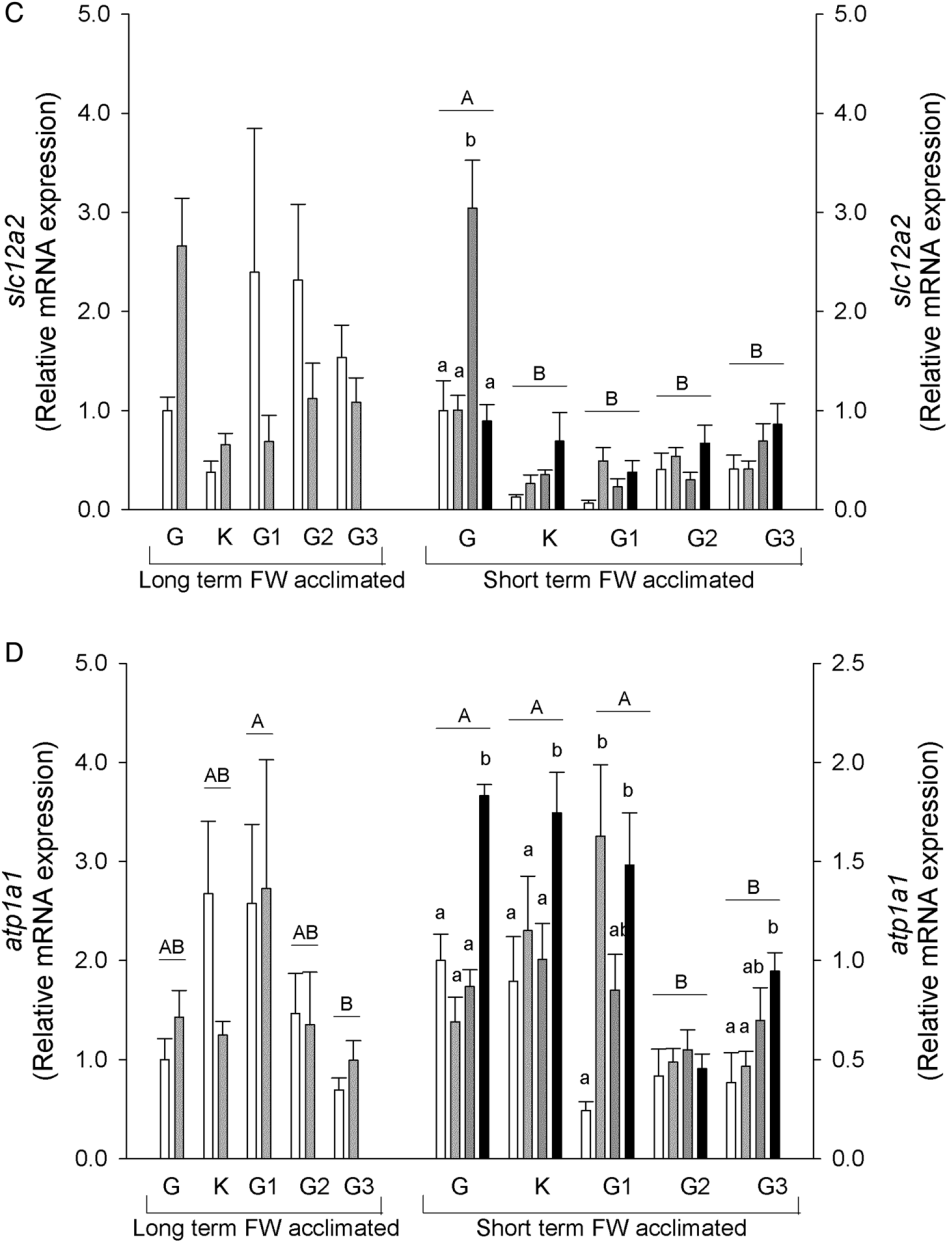

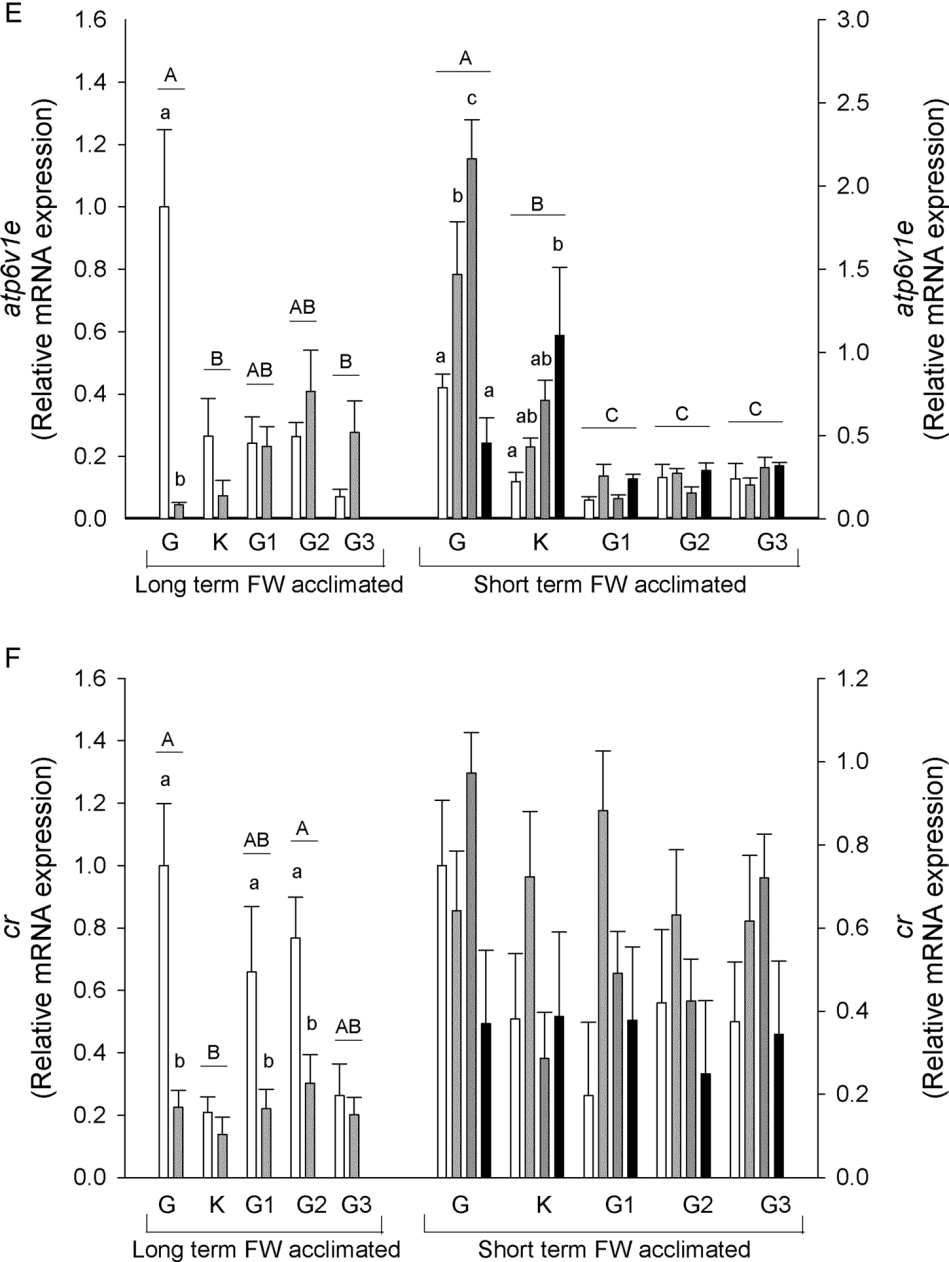
Relative mRNA expression of *scnn1*/ENaC (**A**), *slc12a3*/NCC (**B**), *slc12a2*/NKCC1 (**C**), *atp1a1*/NKA-a (**D**), *atp6v1e*/V-ATPase subunit E (**E**) and corticosteroid receptor (**F**) in the gill, kidney, anterior, middle and posterior intestine of long- and short-term freshwater-acclimated sea lampreys in freshwater and brackish water. See legend to Fig. 1 for details. Values are relative to the respective freshwater control.

Changes at the mRNA level of key ion transport genes (*scnn1*/ENaC, *slc12a3*/NCC, *slc12a2*/NKCC1, *atp1a1*/NKA1a1 and *atp6v1e*/V-type H^+^-ATPase E-subunit) and corticosteroid receptor (*cr*) were examined using a real-time RT-PCR approach (Fig. 2).

The *scnn1* mRNA expression levels were highest in the gill and kidney of long-term FW-acclimated salinity-challenged sea lampreys, with lower levels in the intestine. A decrease in relative *scnn1* mRNA expression was found in the gill, kidney and middle and posterior intestine with salinity challenge. In the short-term FW-acclimated sea lampreys, *scnn1* was highest in the gill and decreased in all salinity groups in this tissue (Fig. 2A). There were no changes in the other tissues, which had lower starting mRNA levels.

The *slc12a3* mRNA levels were highest in the gill in both long- and short-term FW-acclimated lamprey groups and lower in the kidney, with no expression found in the intestinal regions. In the gill, mRNA expression decreased in all salinity-challenged animals. In short-term FW-acclimated lampreys, BW-25 challenged osmocompromised animal *slc12a3* mRNA was markedly lower than all other groups (Fig. 2B).

The *slc12a2* mRNA expression remained unaltered in the long-term FW-acclimated sea lampreys, although significant differences may have been masked by high variation. In short-term FW-acclimated lampreys, higher levels of *slc12a2* mRNA expression were found in the gill compared with all other tissues analysed, and an increase in expression was found in the gill BW-25 osmoregulators (Fig. 2C).

In long-term FW-acclimated lampreys *atp1a1* mRNA expression was found to be highest in the anterior intestine, in contrast to the lowest levels in the posterior intestine, with salinity having no effect on mRNA expression. Short-term FW-acclimated lampreys showed higher *atp1a1* mRNA expression in the gill, kidney and anterior intestine compared with middle and posterior intestine regions. In BW-25 osmocompromised animals, expression increased in all tissues except middle intestine. In BW-17.5 animals, *atp1a1* mRNA also increased in anterior intestine (Fig. 2D).

V- ATPase E subunit (*atp6v1e*) mRNA expression was highest in the gill of long-term FW-acclimated sea lampreys, with its levels decreasing ∼20-fold in animals acclimated to 17.5 BW. No significant differences were observed in the other tissues with salinity challenge. In short-term challenged animals, the highest *atp6v1e* mRNA expression was observed in the gill, followed by the kidney. In the gill, the mRNA expression was upregulated in BW-17.5 and BW-25 osmoregulating animals but not in BW-25 osmocompromised animals. In contrast, in the kidney, BW-25 osmocompromised animals showed significantly upregulated *atp6v1e* mRNA expression, whereas in the other salinity groups the increases were not significant. Expression in intestinal sections was low and remained unchanged (Fig. 2E).

The *cr* mRNA expression was found to be downregulated by salinity in the gill and anterior and middle intestine in the long-term FW-acclimated lampreys. Expression levels were lowest in the kidney and unresponsive to salinity. No changes were found in the short-term FW-acclimated sea lamprey groups (Fig. 2F).

### Immunohistochemistry

**Figure 3:**
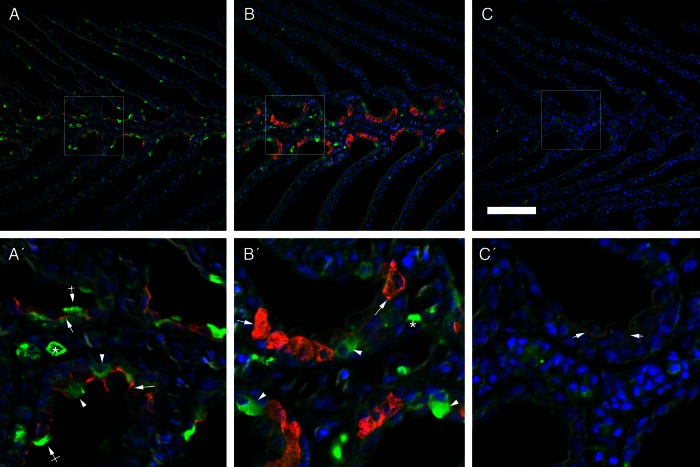
Double immunofluorescence localization of V-ATPase (green) and Na^+^/K^+^-ATPase (red) with the corresponding merged image overlaid with DAPI nuclear staining (blue) in the gills of upstream-migrating lampreys in a freshwater-acclimated lamprey (**A**), a BW-25 osmoregulator (**B**) and a BW-25 osmocompromised animal (**C**). Arrows indicate NKA basolateral immunoreactivity. Arrowheads and crossed arrows indicate V-ATPase epithelial cytosolic and apical staining, respectively, while asterisks indicate leukocyte V-ATPase staining. Scale bar: 100 µm.

Gill sections probed for Na^+^/K^+^-ATPase (NKA) and V-type H^+^-ATPase are shown in Fig. 3. In sea lampreys acclimated to FW, V-ATPase was immunolocalized to isolated epithelial cells in both the filament and the lamellae. Two types of cellular staining patterns were displayed, which included whole-cell signal and some cells showing labelling concentrated more on the apical region of these cells (Fig. 3A). Strongly immunoreactive cells were also observed in the blood space, which were probably leukocytes. In FW animals, NKA immunoreactivity was limited to the basolateral membrane of epithelial cells in the filament but not the lamellar epithelium. When lampreys were acclimated to BW, osmoregulators displayed fewer H^+^-ATPase immunoreactive cells in the lamellae, and a similar immunolocalization pattern was found in the filament epithelium, although there were few cells with apical immunoreactivity. In these conditions, intense NKA immunoreactivity was found in clusters of large cells (two to five cells) in the filament epithelium, with labelling present throughout the cell body indicative of seawater-type ionocyte tubular system staining (Fig. 3B). In BW osmocompromised animals, Na^+^/K^+^-ATPase immunoreactivity was weak and limited to the basolateral membrane of cells in the filament epithelium, and fewer V-ATPase immunoreactive cells were found in the gill with a diffuse cytoplasmic staining pattern (Fig. 3C).

## Discussion

In anadromous migratory species, including the sea lamprey, obstructions to passage, such as dams and weirs, are a major conservation issue (Larinier, 2000; Marmulla, 2001). In Portugal, where this study was conducted, there are dams and other obstructions on the main sea lamprey spawning river systems that result in significant habit loss (Mateus *et al.*, 2012). At best, sea lampreys have been reported to overcome near-vertical obstacles 1.5–1.8 m (Scott and Crossman, 2012), but Reinhardt *et al*. (2009) have shown in laboratory experiments that landlocked sea lampreys cannot climb vertically. If fish passage facilities are available, they are not designed with lampreys in mind and are generally not efficient for lamprey passage (Mateus *et al.*, 2012; Moser *et al.*, 2015). Given that the sea lamprey is a semelparous species, its ability to reach upstream spawning grounds ultimately determines the success or failure for individuals in this closing step of its life cycle (Moser *et al.*, 2015). If unable to overcome an obstacle, the sea lamprey has few options: turning back downstream and finding another FW system in which to spawn; spawning in a suboptimal downstream habitat; or, in the worst-case scenario, dying without spawning. The retreat downstream has been observed in sea lampreys (Applegate, 1950; Kelso and Gardner, 2000; Almeida *et al.*, 2002a; Holbrook, 2015), although these studies are limited to migration in FW. Notably, using telemetry Kelso and Gardner (2000) found that 26% of males (39 of 149) with radio transmitters emigrated (migrated downstream out of the rivers they had been released into) and that there were porportionally more emigrants in rivers with upstream barriers closer to the release point. Holbrook (2015) has also shown that there is a tendency for a small proportion of downstream retreating migrants to keep moving downstream rather than hold or return upstream towards the barrier again. In the Pacific lamprey (*Entosphenus tridentatus*), migrants encountering a barrier have been shown to enter downstream tributaries (Noyes *et al*., 2013). It has been suggested that this behaviour is linked with the search for migratory cues because no strong directional migration is displayed (reviewed by Moser *et al*., 2015).

In the present study, estuarine-captured sea lampreys acclimated to freshwater and then tested for salinity tolerance in March and June were unable to acclimate to full-strength seawater, reaching salinity limits of 25 and 17.5, respectively. This latter value is well correlated with previously reported salinity tolerance levels in upstream anadromous migrants (∼50% seawater; Galloway, 1933; Morris, 1956; Beamish *et al.*, 1978). The short-term FW-acclimated salinity challenged lampreys could be divided into two groups, osmoregulators and osmocompromised animals, which may reflect differences in residency time in the estuary, because lampreys may enter the estuary as early as December.

### Osmocompromised animals

The long-term FW-acclimated lampreys and 18% of the short-term FW-acclimated lampreys could be classified as being osmocompromised and on the verge of osmoregulatory failure. This was marked by increases in plasma ions, with Na^+^, Cl^−^ and Ca^2+^ approaching environmental levels (long-term FW-acclimated lampreys at BW-17.5) or approximately doubling from FW control fish levels (short-term FW-acclimated lampreys at BW-25). These values are in reasonable agreement with the Na^+^ concentrations reported by Beamish *et al*. (1978) given the high variability they observed in their high-salinity treatment (BW-16 and BW-24; plasma [Na^+^] 177 and 226 mM, respectively). As a consequence of these high plasma ion levels, blood haemolysis was observed in the present study, with haematocrit decreasing by >90%. Lewis and Ferguson (1966) have demonstrated that among premammalian erythrocytes, fish erythrocytes are the most fragile using an osmotic fragility test common in haematology. This crash in blood oxygen-carrying capacity would have a implications for the maintenance of aerobic metabolic rates, and osmocompromised animals were observed to be much more sluggish than osmoregulators. Plasma lactate would be an indicator of anaerobic metabolism and has been shown to be negatively correlated with haematocrit (Olsen *et al.*, 1992); however, in the present study no correlation was found and changes were not consistent (decrease in long-term FW-acclimated lampreys and increase in short-term FW-acclimated lampreys). It should be noted that haematocrit did decrease in osmoregulating fish as well, but by only 20%. Cellular damage was not limited to red blood cells, because indicators of liver and muscle damage in fish (Kumari *et al.*, 2011; Gabriel *et al.*, 2012; Gaim *et al.*, 2015) were also increased in plasma (LDH, AST and ALT levels). These cytosolic enzymes are released into the plasma when tissues are damaged (e.g. Vedel *et al.*, 1998; Pakhira *et al.*, 2015). Aspartate aminotransferase is a good predictor of liver damage (Casillas *et al.*, 1982; Murray, 1984), and levels were increased in BW-25 osmocompromised animals. Lactate dehydrogenase and ALT are released when other tissues are damaged, notably muscle (Kumari *et al.*, 2011). Dehydration was also observed in muscle and accompanied by increases in Na^+^ levels. Although muscle water content values in FW in the short-term FW-acclimation study were lower than literature values of ∼77% (Kott, 1971; Boutilier *et al.*, 1993), taken together the impacts on muscle are correlated with the lethargy observed in osmocompromised animals.

In osmocompromised animals, the emergence of seawater-type ionocytes in the gills was not observed. In lampreys, seawater-type ionocytes are readily identifiable by the appearance of whole-cell NKA immunoreactivity, which represents labelling of the tubular membrane system (Bartels and Potter, 2004; Reis-Santos *et al.*, 2008). Unlike teleost fishes, which have this staining pattern in both FW and seawater ionocytes, in lampreys it is associated only with the seawater-type ionocytes (Wilson, 2011). Only the NKA immunoreactivity pattern of basolateral staining in filament cuboidal pavement cells observed in FW lampreys was present in osmocompromised animals (Choe *et al.*, 2004; Reis-Santos *et al.*, 2008). Also, no transcriptional upregulation of gill ion transporters NKCC1 and NKA was observed. Taken together, these results indicate a failure of a hyposmoregulatory branchial response in osmocompromised animals.

In the gut, there were generally few changes in ion transporter transcript levels that would indicate a hyposmoregulatory response (Li *et al.*, 2014). It has previously been shown that drinking rates decrease upon FW acclimation, and thus, switching drinking back on in salinity-challenged animals probably limits complete seawater tolerance (Rankin, 2002). Also, the start of their spawning migration and the development of the gonads are associated with atrophy of the gut and the cessation of feeding (Larsen, 1980). Intestinal fluid Cl^−^ concentrations doubled in osmocompromised animals when compared with the respective FW control group, from almost iso-ionic with plasma in FW and BW-25 osmocompetent animals. This high intestinal Cl^−^ suggests that drinking is occurring but that oesophageal desalination has failed, and thus, water absorption is compromised (Loretz, 1995).

### Osmoregulators

Given that animals challenged in March were able to osmoregulate in salinities of 25, returning to estuarine waters becomes physiologically possible. The upregulation of branchial NKCC1 mRNA in the short-term FW-acclimated lampreys and the appearance of characteristic NKA-immunoreactive seawater-type ionocytes in the gill epithelium indicate a hyposmoregulatory response to support the ability of the fish to ionoregulate. Morris (1958) has also shown that a small percentage of freshly caught, migrating river lampreys (*Lampetra fluviatilis* L.) were able to osmoregulate in 50% seawater, osmoregulating in a similar manner to marine teleosts. The kidney would produce low volumes of highly concentrated urine, and the water-swallowing mechanism would be responsible for compensating the loss of water at the body surface and Cl^−^ excreted by the seawater ionocytes (Morris, 1958).

### Successful downregulation of freshwater ion-uptake mechanisms

Regardless of whether lampreys could successfully hyposmoregulate in BW or not, all were clearly able to downregulate branchial ion-uptake mechanisms (ENaC and NCC); however, the responses of branchial ion pumps NKA and V-ATPase were ambiguous. In the case of the NKA, given its dual importance in both active ion uptake and secretion (McCormick, 1995; Reis-Santos *et al.*, 2008), this is not entirely unexpected. In the case of the V-ATPase, which is associated with indirect coupling of Na^+^ uptake via the ENaC, decreases in long-term FW-acclimated lampreys were as expected; however, in short-term FW-acclimated lampreys the transcript expression increased significantly. The significance of this increase is unclear, although it may be associated with increased leukocyte infiltration into gill tissue. Also, immunohistochemistry indicates that apical expression of V-ATPase is lost, suggesting that although present in the gill it is not functional in Na^+^ uptake.

In the kidney, the switch from hyper- to hyposmoregulation is associated with a decrease in NKA expression (Lin *et al.*, 2004), and this was apparent in adult sea lampreys (mRNA, protein and activity). In the colon of mammals, ENaC is involved in Na^+^ reabsorption (Malsure *et al.*, 2014), and in the present study, in the middle and posterior intestine, ENaC transcript expression decreased significantly in long-term FW-acclimated lampreys, suggesting an adaptive response.

Although 11-deoxycortisol has been reported to be the mineralocorticoid in lampreys (Close *et al.*, 2010), corticosteroid receptor mRNA expression was not affected by salinity in the short-term FW-acclimated lamprey challenges, although in long-term FW-acclimated lamprey challenges, downregulation was found in the gill and anterior and middle intestine.

### Conclusions

Given that very little is known about osmoregulatory mechanisms in estuarine and migrating adult lampreys (Moser *et al.*, 2015), the present study should contribute significantly to this knowledge gap. The anadromous spawning migration phase in the sea lamprey life cycle is a time of profound morphological and physiological change. This study demonstrates that they possess the branchial mechanisms for hyperosmoregulation (e.g. ENaC and NCC) and, although they can successfully downregulate these mechanisms upon salinity re-exposure following FW acclimation, their ability to conversely upregulate hyposmoregulatory mechanisms can be limiting. It has been shown that it is physiologically possible for lampreys to return to salinities of 25 and, although downstream retreat (emigration) has been observed in lampreys that encounter barriers to upstream migration, it remains to be determined whether anadromous lampreys will in fact retreat to the estuarine or coastal waters in search of suitable spawning grounds.

## Supplementary material

Supplementary material is available at *Conservation Physiology* online.

## Funding

This work was partially supported by the European Regional Development Fund (ERDF) through the Competitiveness and Trade Expansion Program (COMPETE) and by National Funds provided by Fundação para a Ciência e a Tecnologia (FCT) via the research project PTDC/MAR/98035/2008 and European Regional Development Fund through the COMPETE – Operational Competitiveness Program and national funds through FCT [PEst-C/MAR/LA0015/2011] to J.M.W.

## Supplementary Material

Supplementary Data
