# Supplementary material for: Effects of salinity on upstream-migrating, spawning sea lamprey, Petromyzon marinus
Source: Conserv Physiol. 2016 Feb 6;4(1):cov064. doi: 10.1093/conphys/cov064 (PMC4765514; doi:10.1093/conphys/cov064)
Supplement: Supplementary Data [file supp_4_1_cov064__index.html]

Effects of salinity on upstream-migrating, spawning sea lamprey, Petromyzon marinus — Supplementary Data 

# Effects of salinity on upstream-migrating, spawning sea lamprey, *Petromyzon marinus*

## Supplementary Data

Supplementary Data

- Supplementary Data - Docx file
