# Supplementary material for: Effects of salinity on upstream-migrating, spawning sea lamprey, Petromyzon marinus
Source: Conserv Physiol. 2016 Feb 6;4(1):cov064. doi: 10.1093/conphys/cov064 (PMC4765514; doi:10.1093/conphys/cov064)
Supplement: Supplementary Data [file supp_cov064_cov064supp.docx]

**Supplementary materials**

Supplementary material Table 1 Primer pairs (sense and anti-sense, respectively) for qPCR with predicted product size, annealing temperature, original gene accession number. Primers with an asterisk (*) were designed with Primer3 and reference sources are given for the remainder.

| **Gene name** | **GenBank Accession No.** | **Forward and reverse primer sequences (5’-3’)** | **Product size (bp)** | **Annealing temperature (ºC)** | Reference |
| --- | --- | --- | --- | --- | --- |
| *gapdh* | AY578058 | TGCAAAGCACGTCATCATCTC TTCTCGTGGTTTACTCCCATCA | **72** | **60** | (Shifman *et al.*, 2009) |
| *atp1a1** | GENSCAN00000136072 | CGTGGAATCGTCATCAACAC  GCGACAGGATGAAGAAGGAG | **169** | **58** |  |
| *slc12a2** | [ENSPMAG00000000665](http://www.ensembl.org/Petromyzon_marinus/Gene/Summary?db=core;g=ENSPMAG00000000665;r=GL479336:3629-33813;t=ENSPMAT00000000732) | GAGAGGTTTCGCGACAAGAC  CGCTCACGAGTAGAACGTCA | **225** | **58** |  |
| *slc12a3** | ENSPMAG00000005880 | GTCATCACGGTCACCTTCCT  ACACCGGAGTGAAATTCTCG | **205** | **58** |  |
| *atp6v1e** | ENSPMAG00000008972 | GTGAAGGAAGCCATGGAGAA  TGGGGTTGACTTTGAAGAGC | **232** | **58** |  |
| *scnn1** | [ENSPMAG00000007655](http://www.ensembl.org/Petromyzon_marinus/Gene/Summary?db=core;g=ENSPMAG00000007655;r=GL482990:2869-9839;t=ENSPMAT00000008481) | GCATCATGGTACACGACCAG  AGGCGGAGGAGTAGAGGTTC | **183** | **58** |  |
| *cr** | [AY028457.1](http://www.ncbi.nlm.nih.gov/nucleotide/13919633?report=genbank&log$=nucltop&blast_rank=1&RID=UXG5Y9BA013) | GTCCCACAAGAGGGTCTGAA  GGCCATCATGTCAGGAAACT | **247** | **60** |  |
